# Presence of *Candida Albicans* in Root Canal System of Teeth Requiring Endodontic Retreatment with and without Periapical Lesions

**Published:** 2007-04-01

**Authors:** Hengameh Ashraf, Mohammad Samiee, Gita Eslami, Mohammad Reza Ghodse Hosseini

**Affiliations:** 1 *Department of Endodontics, Dental School, Shahid Beheshti University of Medical Sciences, Tehran, Iran*; 2 *Department of Microbiology, Medical School, Shahid Beheshti University of Medical Sciences, Tehran, Iran*; 3 *Endodontist, Tehran, Iran*

**Keywords:** Candida Albicans, Endodontic Treatment, Microorganism, Periapical Lesion

## Abstract

**INTRODUCTION:** An important consideration in endodontic treatment is the elimination of microorganisms, including fungi, from the complex three- dimensional root canal system. *Candida Albicans* (CA) has a major role in endodontic treatment failure as the most important fungus isolated from the root canal system. The present study was carried out to evaluate the presence of CA in the teeth requiring endodontic retreatment, with or without periapical lesions.

**MATERIALS AND METHODS:** This study was carried out on 60 root canals from human molars requiring endodontic retreatment. The root canals were randomly divided into two equal groups of 30 canals with versus without periapical lesions. Samples were collected from the root canals and cultured on MacConkey and blood agar culture media. The samples suspected of having CA were streaked on Sabouraud’s Dextrose Agar (SDA) and evaluated under a light microscope. Data was analyzed and compared using Chi- square and Kappa tests.

**RESULTS:** CA was found in 11 patients (36.7%) with periapical lesions. In the patients without periapical lesions only 4 samples demonstrated CA in the root canal systems (13.3%). The difference between the two groups as to the presence of CA was statistically significant (p<0.037). In addition, evaluation of salivary samples revealed 15 cases (50%) of CA presence in the patients with periapical lesions and 16 cases (53.3%) of CA in the patients without periapical lesions, demonstrating no statistically significant difference between the two groups.

**CONCLUSION:** Considering the fact that the presence of CA in the root canal systems of teeth with periapical lesions was more noticeable and statistically significant compared to the teeth without periapical lesions, the elimination of this microorganism from the root canal system, using appropriate intracanal solutions and medications is of utmost importance.

## INTRODUCTION

Some studies have evaluated the presence of *Candida Albicans* and other microorganisms in teeth with or without periapical lesions. An important consideration in endodontic treatment is elimination of microorganisms from the complex three-dimensional root canal system, especially from the dentinal tubules. Root canals function as an incubator since they are closed spaces with low oxygen concentration and therefore they promote the growth of microorganism. On the other hand, it has been demonstrated that fungi have a role in endodontic treatment failure and CA has a greater role in the failure than others (1). As a result of limited access to different regions in the root canal system, preparing a canal completely devoid of microorganisms in an infectious tooth is extremely difficult or even impossible despite proper Instrumentation and irrigation (1,2). Resistant microorganisms such as *Enterococcus Faecalis*, gram-negative facultative anaerobic bacilli and more importantly Pseudomonas can have a major role in the persistence of infection in the root canal system (1-4).

Studies have demonstrated that fungi are also present in infections resistant to conservative root canal treatment and despite proper cleaning and irrigation of the root canal system play a role in failure of periapical lesions treatment (5-7).

CA is a pleomorphic microorganism and the variable growth patterns of its different parts give rise to its various components such as blastospores and chlamydospores etc (8).

Conversion of CA from an innate micro-organism to a pathogenic one depends on minor changes in varius pathogenic characteristics such as adhesion factor (thigmotropism), Hypha formation, proteinase secretion and phenotypic switching phenomenon (9).

Endodontic treatment success, to great extent, depends on the depletion or elimination of microorganisms from the root canal system. To this end, the use of irrigating solutions with proper anti-microbial and anti-fungal properties during canal debridement and preparation are considered enormously important (1).

Waltimo *et al*. introduced fungi as micro-organisms resistant to endodontic treatment in apical periodontitis and demonstoated that CA species requires incubation with a saturated solution of calcium hydroxide for 16h (10). In that study 48 fungal types were isolated from 47 specimens (7%) out of 692 chronic apical periodontitis cases resistant to endodontic treatment.

Peciuliene *et al*. isolated fungi as resistant microorganisms in the obturated root canals of teeth with chronic apical periodontitis (11). A study carried out by Egan *et al*. which aimed at evaluating the relationship between the presence of fungi in the saliva and in the root canals of teeth with apical periodontitis, revealed that fungi were 13.8 times more prevalent in the root canals when they were also present in the salivary samples of the subjects (12). The study established a clear relationship between the presence of fungi in the root canal system and their presence in saliva. That study, which evaluated 60 root canals and salivary samples from the subjects revealed that CA was the most prevalent fungus isolated (13). The presence of CA in the microbial population of root canals and in persistent lesions has prompted researchers to test new (intracanal) medications and methods to eliminate this fungs from the root canal system. The effect of varying concentrations of MTA on CA was evaluated (13).

In addition, other intra-canal medications such as Ca(OH)2 and chemicals that can be used with Ca(OH)2 to eliminate CA from the root canal system are currently under investigation (14-16).

On the other hand, apical periodontitis is an inflammatory process in the periapical region and in most cases chemomechanical prepara-tion with intra-canal medications such as Ca(OH)2 followed by canal obturations with gutta-percha and a sealar results in the resolution of the infection and the repair of the periapical lesions. However, in some cases apical periodontitis does not properly respond to root canal treatment and leads to periapical infection, persisting for months or even years after routine root canal treatment (13-16).

The present study was carried out to evaluate the presence of CA in the teeth requiring endodontic retreatment, with or without periapical lesions, considering the importance of the subject in clinical situations and the necessity of the elimination of CA from dentinal tubules.

## MATERIALS AND METHODS

The current cross-sectional analytical study was carried out on 60 canals from maxillary and mandibular molars requiring endodontic retreatment. The subjects were selected by simple non- random method from those referring to the Endodontic Department of Shahid Beheshti Dental School. The teeth had received endodontic treatment in private dental clinics and no other information was available regarding the teeth. Subsequent to clinical examination and evaluation of existing restorations and taking radiagraphs, the teeth with fractured restorations or with caries along with coronal leakage were excluded from the study.

The tools for data collection included observation and microbiologic cultivation in a laboratory. The patients did not suffer from any co-existing systemic conditions such as diabetes, leukemia, anemia, immuno-deficiency etc. In addition, their medical history did not reveal any long-term use of antibiotics or corticosteroids or any medications which contribute to the elimination of Candida species. The root canals were randomly divided into two equal groups of 30, one group with periapical lesions and the other without periapical lesions and as far as it was possible the two groups were matched. For the differential diagnosis of the lesions radiographs were taken using differing X- ray angulations.

Subsequent to case selection and before collecting samples from the root canals a salivary sample was collected from each patient using sterile swabs. Then, each tooth under study was polished using a polishing cup and isolated with rubber dam. Tooth surroundings were irrigated with 10% iodine followed by cleansing with normal saline. Subsequent to the removal of existing restorations, the procedures to eliminate contamination were repeated. To avoid the contamination of samples with microorganisms, pre-existing gloves were removed and new sterile surgical gloves were worn during sample collection. At first, gutta-percha was removed from the coronal thirds of canals with periapical lesions using # 2 Gates- Glidden drills. Subsequently, samples were collected from gutta-percha and the canal walls in the middle and apical thirds of the canals using #20 Hedstrom files (Dentsply, Maillefer, Switzerland).

In order to scrape canal walls Hedstrom files were placed in the canals to the working length and were moved on the canal walls to remove sufficient amounts of dentin out of the canals and into the culture media. The collected salivary and canal samples were immediately transferred into the transport medium, namely TS Broth (Merck, Merck, Germany), in such a manner that cutting edges of the files were completely immersed in the medium. At a maximum of 3h after sample collection, the samples were sent to the microbiology laboratory in Shahid Beheshti Medical School.

The samples were cultured on two primary culture media consisting of blood agar (Merck, Merck, Germany) and MacConkey agar (Merck, Merck, Germany). Twenty-four hours after placing the culture media in a CO2 incubator, only salivary samples demonstrated microorganism growth. Therefore, to allow for the better growth of the canal samples, they were placed in a CO2 incubator (for another 24h) and after removal from the incubator the samples were cultured on two primary culture media of blood agar (Merck, Merck, Germany) and MC (Merck, Merck, Germany).

Subsequently, the cultures were placed for another 24h in a CO2 incubator (Bat tech, LCO- 263 AI, Korea).

In the patients with periapical lesions, 15 samples were collected from the maxillary molars and 15 others were collected from the mandibular molars; in the patients without periapical lesions, 13 samples were collected from the maxillary molars and 17 samples were collected from the mandibular molars.

Twenty-Four hours after the primary cultivation, the grown colonies of micro-organisms were classified based on their type, morphology and Gram-staining and were transferred to differential culture media. The differential culture media were selected according to the types of microorganisms on microscope slides and diagnostic test; then the samples were cultured.

Twenty-Four hours after the primary cultivation and gram-staining and the observation of dark pink yeasts, the samples suspected of CA presence were transferred to the special culture media of SDA (Merck, Merck, Germany) , which is a special rich medium for CA growth. The samples were streaked on the plates and the culture media were subsequently kept at 37°C for 24h. The presence or absence, color, morphology and number of the grown *Candida* colonies were evaluated and confirmed under a light microscope. The collected data was analyzed and compared using SPSS software and Chi-square and Kappa tests.

## RESULTS

The results demonstrated CA in the root canals in 11 samples (36.7 %) out of 30 samples in the patients with periapical lesions; 5 cases were from the maxilla and 6 others were from the mandible. Only 4 cases from the patients without periapical lesions demonstrated CA in the root canals (13.3%); 2 cases were related to the maxilla and 2 cases were from the mandible, statistical analysis of data using Chi-square test demonstrated significant difference between the patients with and without periapical lesions (p<0.037). Evaluation of salivary samples revealed 15 cases (50%) of CA presence in the patients with periapical lesions and 16 cases (53.3 %) of CA in the patients without periapical lesions, demons-trating no statistically significant difference between the two groups.

Evaluation of the relationship between the presence of CA in the saliva and its presence in the root canals in the two groups did not reveal any statistically significant relationship, in the subjects with periapical lesions Kappa was calculated at 0.20 (p>0.25). In the subjects without periapical lesions Kappa was calculated at 0.017 (p>0.88).

## DISCUSSION

The present study revealed that the odds of CA presence in the teeth with periapical lesions, requiring endodontic retreatment (36.7%) is more than that in the teeth without periapical lesions, requiring endodontic retreatment (13.3%), indicating a statistically significant difference.

According to the results of the present study CA is the most prevalent fungus in the root canal system. Siqueria and Sen, in a study on the role of fungi in endodontic failure, reported that CA is the most prevalent fungus in such cases, which is consistent with the results of the present study (17). Furthermore, the results of a study carried out by Waltimo *et al*. revealed the presence of fungi in 5% - 20% of infected root canals concomitant with apical periodontitis (18).

The differences between the results of these two studies may be attributed to different geographical locations and differences in the oral hygiene habits. In these two studies virulence factors of fungi, including adaptability with different environmental conditions, adhesion to different surfaces, the secretion of proteolytic enzymes, biofilm production, morphologic changes and the stimulation of immune and defense mechanisms of the host, were mentioned, which have a role in the pathogenicity of fungi in periradicular lesions.

In a study carried out by Egan *et al.* the odds of the CA presence in the root canals with apical periodontitis concomitant with a positive saliva test for CA was 13.8 times higher than a situation in which the saliva test was negative for CA (12). According to the results of that study fungi were present in 10% of the root canals and there was a meaningful relationship between the presence of fungi in the root canals and their presence in saliva. In the present study, the presence of CA in the root-filled teeth with and without periapical lesions was confirmed and its presence in salivary samples was demonstrated.

In a study carried out by Waltimo *et al*. on 967 microbiologic endodontic samples 48 types of fungi were isolated from 47 samples and all the samples except one contained *Candida* genome (10).

A review of the results of various studies reveals the predominance of CA in the fungal samples. The samples in the present study were obtained by filing the dentinal walls of the canals and the presence of CA was confirmed after culturing the samples in the specialized SDA therefore, CA can be considered a dentinophilic microorganism. Also, in a study carried out by Sen *et al*. CA was reported to be the most prevalent pathogenic fungus isolated from the oral cavity and was called a dentinophilic microorganism (19).

In a study carried out by Najzar-Fleger *et al*. carious lesions were reported the main source of fungus presence and dental caries was reported the only part of entry of fungi into the root canal system (20). However, in the present study CA was found in 25% of root-filled canals; this percentage of CA presence and the attempt, in this study, to exclude any carious, fractured and coronally leaking teeth from the study may be the reasons to attribute the presence of these microorganisms to the lack of proper and complete isolation of the teeth during endodontic treatment. Therefore, proper isolation of teeth during endodontic treatment may prevent microorganisms, including CA, from entering the root canal system.

## CONCLUSION

According to the results of present study, the presence of CA in the root canal system of teeth with periapical lesions was more noticeable and statistically significant compared to the teeth without periapical lesions, necessitating its elimination from the root canal system using appropriate intracanal medications and irrigating solutions.
